# Lifestyle Modify Optic Nerve Injury in Mendelian Randomization

**DOI:** 10.1155/genr/6698323

**Published:** 2025-12-25

**Authors:** Maobin Zhou, Yanyu Shangguan, Xiaodong Liu

**Affiliations:** ^1^ Department of Orthopedics, Yangpu Hospital, Tongji University School of Medicine, Shanghai, 200090, China, tongji.edu.cn; ^2^ Department of Ophthalmology, Tongji Hospital, Tongji University School of Medicine, Shanghai, 200065, China, tongji.edu.cn

**Keywords:** fibrosis, lipid metabolism, Mendelian randomization study, optic nerve injury, physical activity

## Abstract

**Background:**

Optic nerve injury, as a neurodegenerative disorder, leads to irreversible visual impairment. Although the underlying mechanisms linking physical activity to optic nerve injury remain unclear, this study aimed to establish a causal relationship between physical activity and optic nerve injury using a Mendelian randomization (MR) framework.

**Methods:**

This MR study utilized genome‐wide significant variants as instrumental variables (IVs) for assessing the relationship between physical activity and optic nerve injury, focusing on individuals of European descent. The approach was supported by comprehensive sensitivity analyses and augmented by bioinformatics tools including differential gene expression, Gene Ontology (GO), and Kyoto Encyclopedia of Genes and Genomes (KEGG) enrichment analyses.

**Results:**

Our study demonstrated that after adjustment for MVPA, alcohol intake, BMI, blood glucose, blood lipids, and smoking, LST was positively associated with glaucoma risk (*β* = 0.016, 95% CI: 0.004 to 0.027, *p* = 0.014), indicating its role as an independent risk factor. Conversely, MVPA was negatively associated with glaucoma risk (*β* = −0.012, 95% CI: −0.022 to −0.002, *p* = 0.026), supporting a protective effect, while smoking also showed a significant association (*β* = −0.020, 95% CI: −0.039 to −0.002, *p* = 0.037). Sensitivity analyses confirmed the robustness of these findings, and bioinformatic analyses implicated cholesterol metabolism and fibrosis pathways in optic nerve injury.

**Conclusion:**

These findings underscore the potential of lifestyle modifications, including increased physical activity and reduced sedentary behavior, as a cost‐effective strategy to prevent and manage optic nerve injury.

## 1. Introduction

Optic nerve injury is an irreversible neurodegenerative disorder that commonly leads to visual impairment and even blindness [1]. This condition not only profoundly diminishes patients’ quality of life but also imposes a considerable burden on global healthcare systems [2]. Pathologically, optic neuropathies can be broadly categorized into three groups: (i) inflammatory optic neuritis induced by viral, bacterial, or fungal infections; (ii) optic nerve congestion and edema associated with optic disc swelling, as seen in glaucoma; and (iii) structural damage to the optic nerve, most frequently presenting as optic atrophy [3]. Consequently, investigating the pathogenesis of various forms of optic nerve injury is essential for developing early intervention and preventive strategies. These efforts are essential for reducing the incidence of optic neuropathies and slowing the progression of vision loss. Such goals are consistent with the World Health Organization’s call for “integrated, people‐centered eye care” aimed at preventing blindness and visual impairment [4].

Emerging evidence indicates that modifiable lifestyle factors, particularly the frequency of physical activity (PA), influence the risk of optic nerve injury in both healthy individuals and patients [5]. Low‐energy wakeful behaviors (≤ 1.5 MET), including sedentary activity and excessive sleep, may accelerate neurodegeneration by promoting systemic inflammation and vascular dysfunction [6]. In contrast, low‐to‐moderate intensity endurance exercise has been shown to reduce intraocular pressure, a well‐established risk factor for optic nerve damage, thereby providing a potential nonpharmacological strategy for disease prevention and intervention [7]. However, several studies have failed to establish a protective association between PA and optic nerve injury in healthy populations, suggesting that the benefits of PA may be particularly evident only among individuals with high genetic susceptibility to such injuries [8, 9]. No studies have ever reported on the potential gene–environment interactions involving PA and optic nerve damage. Although the level of PA is hypothesized to modulate the severity or progression of optic nerve injury, inherent limitations of observational studies, such as limited sample sizes, residual confounding, and misclassification [10], nevertheless preclude robust causal inference regarding this relationship [11].

Previous Mendelian randomization (MR) studies have investigated the relationship between lifestyle factors and glaucoma; however, they have not comprehensively addressed its impact on the essential nature of the optic nerve from a global perspective of optic nerve injury [12]. Furthermore, these studies did not explore the global genetic correlations or underlying mechanisms among relevant traits. Therefore, a more in‐depth investigation into the genetic correlations, causal pathways, and potential mechanisms of these associations is warranted. Building upon genome‐wide association studies (GWASs) and linkage disequilibrium score regression (LDSC), we applied MR by using single‐nucleotide polymorphisms (SNPs) as instrumental variables (IVs) to infer causal relationships between exposures and outcomes [13–15]. This approach minimizes confounding inherent in conventional epidemiological studies. Using this analytical framework, we sought to clarify the causal associations of sedentary behavior, sleep, and PA with optic nerve health. This study innovatively considers multiple types of optic nerve injury as a unified phenotype, revealing novel mechanisms through which lifestyle factors influence optic nerve health from a global genetic perspective. This approach overcomes the limitations of previous studies that were confined to single disease entities.

## 2. Methods

### 2.1. Overall Study Design

This study employed univariable MR (UVMR) and multivariable MR (MVMR) approaches, leveraging SNPs from GWAS as IVs, to examine the causal relationships between sedentary behavior, PA, and traits associated with optic nerve injury. Sensitivity analyses were subsequently conducted for significant findings. The MR analysis adhered to three core assumptions: (a) a strong association between the IVs and the exposure; (b) independence of the IVs from known confounders; and (c) influence of the IVs on optic nerve injury solely through the exposure [16].

**Figure 1:**
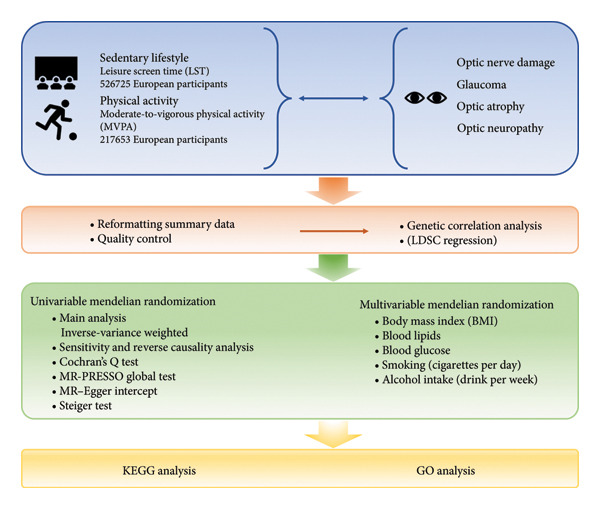
Flow diagram of study design.

LDSC was performed to estimate global genetic correlations. We then applied the MR–CAUSE framework, which uses summary‐level effect estimates, to evaluate the fit of models representing either genetic sharing or causal pathways. Finally, Kyoto Encyclopedia of Genes and Genomes (KEGG) and Gene Ontology (GO) enrichment analyses were conducted to elucidate the underlying biological pathways. A detailed workflow of the analytical procedures is presented in Figure 1.

### 2.2. Data Sources

The genetic IVs used in this study were derived from publicly available summary statistics of large‐scale GWAS. Genetic data for glaucoma were obtained from the UK Biobank study, comprising 462,933 individuals of European ancestry (OpenGWAS ID: ukb‐b‐8398). Data for optic atrophy were sourced from a large‐scale genetic association study by Zhou et al. [17], including 401,303 European‐ancestry participants. Genetic instruments for optic neuropathy were obtained from the Finnish FinnGen database, with a sample size of 450,415 individuals.

For exposure variables, genetic instruments for leisure sedentary time were derived from a systematic genetic investigation by Wang et al. [18] on PA and sedentary behavior, which conducted GWAS on multiple activity‐related phenotypes (*N* = 526,725). Instruments for moderate‐to‐vigorous PA (MVPA) were obtained from a mixed‐linear model association study by Jiang et al. [19] designed for biobank‐scale data (*N* = 217,653).

All data were derived exclusively from individuals of European ancestry. Each dataset used in this study was approved by the respective ethics review boards, and informed consent was obtained from all the participants.

### 2.3. Selection of IVs

SNPs significantly associated with LST and MVPA were selected as IVs for the MR analysis. The validity of these IVs was evaluated according to the three core assumptions of MR: (i) relevance: the genetic variants must be robustly associated with the exposure of interest; (ii) independence: the selected variants should not be associated with potential confounders; and (iii) exclusion restriction: the genetic instruments should affect the outcome only through the exposure, with no alternative pathways.

The significance threshold for SNPs associated with LST and MVPA was set at *p* < 10^−6^, with this threshold selection referring to standard practices in large‐scale GWAS of behavioral traits [20]. To ensure independence among instruments, a linkage disequilibrium (LD) threshold of 10,000 kb and *r* ^2^ < 0.1 was applied during clumping. Palindromic SNPs were removed. Furthermore, SNPs showing significant associations with the outcome were excluded based on the outcome GWAS summary statistics. To mitigate weak instrument bias, we calculated the proportion of variance explained (PVE) and the F‐statistic, retaining only SNPs with an *F* > 10 to ensure robust instrument strength. To address horizontal pleiotropy, MR‐Pleiotropy RESidual Sum and Outlier (MR‐PRESSO) [21] and radial MR methods were employed. Outliers were iteratively removed until the MR‐PRESSO global test yielded a nonsignificant result (*p* > 0.05).

### 2.4. Statistical Analysis

A two‐sample MR framework was employed to evaluate the causal associations between LST, MVPA, and three ocular disorders. First, UVMR analysis [22] was conducted to initially estimate the independent effect of each exposure on each outcome. The inverse‐variance weighted (IVW) [23] method served as the primary analytical approach, providing the most efficient causal estimate under the assumption of no pleiotropy. To verify the robustness of the results, weighted median and MR‐Egger regression methods were additionally applied. The weighted median method yields consistent estimates even when up to 50% of the IVs are invalid, while MR‐Egger regression was used to detect and adjust for potential pleiotropic bias.

We further performed MVMR to simultaneously incorporate LST, MVPA, and other potential confounding factors (including BMI, blood glucose, blood lipids, daily cigarette consumption, and weekly alcohol intake) into the model. The MV‐IVW method served as the primary analytical approach, as it enables estimation of the independent causal effect of the target exposure after adjusting for other related exposures. MVMR analysis helped effectively control for confounding biases arising from correlations between genetic instruments and mutual influences among exposures.

A series of sensitivity analyses were performed to assess the validity and robustness of the MR results. Heterogeneity across IVs was evaluated using Cochran’s *Q* test, with significant heterogeneity (*p* < 0.05) indicating the need for a random‐effects IVW model. Horizontal pleiotropy was assessed by examining the intercept term from MR‐Egger regression, where an intercept with *p* > 0.05 suggested minimal pleiotropic bias. To further evaluate the stability of the findings, leave‐one‐out analysis was conducted by sequentially excluding each SNP to determine whether the overall association was driven by a single variant. In addition, the I^2^GX statistic derived from MR‐Egger regression was calculated to assess instrument strength and its impact on the reliability of the pleiotropy test.

All statistical analyses were conducted using R software (Version 4.3.0) with the “TwoSampleMR” and “MendelianRandomization” packages. Effect estimates are presented as *β* coefficients with 95% confidence intervals. *p* < 0.05 was considered statistically significant.

### 2.5. KEGG and GO Pathway Analyses

SNPs significantly associated with exposure to LST and MVPA were selected, and overlapping genes linked to optic neuropathy–related outcomes were identified. These genes were subsequently submitted to the DAVID database (https://david.ncifcrf.gov/home.jsp) for GO and KEGG pathway enrichment analyses.

## 3. Results

### 3.1. SNP Selection

Following the analytical pipeline, which included clumping, data harmonization, and exclusion of SNPs with an *F* < 10, we identified 115 SNPs associated with LST and 87 SNPs associated with MVPA.

### 3.2. Results of UVMR and Genetic Correlation Analyses

This study employed both UVMR and LDSC to evaluate the associations between LST, MVPA, and three ocular disorders from causal and genetic correlation perspectives, respectively.

UVMR analysis (Figure 1) indicated a positive yet nonsignificant association between LST and the risk of glaucoma (*β* = 0.09, 95% CI: −0.216 to 0.396, *p* = 0.563). Similarly, no significant associations were observed between LST and optic atrophy (*β* = −0.27, 95% CI: −0.77 to 0.23, *p* = 0.297) or optic neuritis (*β* = 0.09, 95% CI: −0.22 to 0.40, *p* = 0.563). For MVPA, a null association was found with glaucoma (*β* = 0.00, 95% CI: −0.01 to 0.00, *p* = 0.563). MVPA showed a positive but imprecise association with optic atrophy (*β* = 0.30, 95% CI: −1.10 to 1.69, *p* = 0.675) and a weak inverse association with optic neuritis (*β* = −0.19, 95% CI: −1.05 to 0.67, *p* = 0.669).

Genetic correlation analyses using LDSC were consistent with the UVMR results (Table 1). LDSC revealed a genetic correlation (rg) of 0.09 (SE = 0.094, *p* = 0.928) between LST and glaucoma, −0.035 (SE = 0.095, *p* = 0.711) between LST and optic atrophy, and 0.031 (SE = 0.095, *p* = 0.745) between LST and optic neuritis. For MVPA, a positive genetic correlation was suggested with glaucoma (rg = 0.126, SE = 0.108, *p* = 0.244) and a weak positive correlation with optic atrophy (rg = 0.05, SE = 0.108, *p* = 0.643), while a negative correlation trend was observed with optic neuritis (rg = −0.107, SE = 0.108, *p*0.326) (*p* > 0.05).

**Table 1:** Genetic correlation between LST, MVPA, and optic nerve injury–related traits estimated using LDSC.

Exposure	Outcome	LDSC		
		rg	se	*p*
LST	Glaucoma	0.09	0.094	0.9279
	Optic atrophy	−0.035	0.095	0.7114
	Optic neuritis	0.031	0.095	0.745

MVPA	Glaucoma	0.126	0.108	0.2438
	Optic atrophy	0.05	0.108	0.6425
	Optic neuritis	−0.107	0.108	0.3256

Sensitivity analyses demonstrated that effect estimates from the weighted median and MR‐Egger methods were generally aligned in direction with those from the IVW approach (Table 2). No significant heterogeneity was detected via Cochran’s *Q* test (*p* > 0.05), and the intercept term in MR‐Egger regression indicated no evidence of horizontal pleiotropy (*p* > 0.05). Leave‐one‐out analysis confirmed that no single SNP disproportionately drove the overall results.

**Table 2:** Sensitivity analysis and MR pleiotropy test of LST, MVPA on glaucoma, optic atrophy, and optic neuritis.

Exposure	Outcome	Heterogeneity_IVW_*P*	Pleiotropy MR‐Egger	Pleiotropy Egger intercept *p*	MR‐PRESSO global test *p*	Steiger test *p*
LST	Glaucoma	0.08627083	−5.69*E* − 05	0.6201697	0.091	0
	Optic atrophy	0.60719754	5.05*E* − 03	0.8746379	0.566	0
	Optic neuritis	0.56133954	−3.67*E* − 03	0.851554	0.53	0

MVPA	Glaucoma	0.001232726	8.05*E* − 05	0.3051796	0.005	7.32*E* − 223
	Optic atrophy	0.520612934	1.43*E* − 02	0.4623036	0.522	5.95*E* − 231
	Optic neuritis	0.437607591	−1.72*E* − 02	0.151272	0.439	6.22*E* − 230

In summary, both UVMR and genetic correlation analyses did not reveal significant causal associations or genetic architectures linking either LST or MVPA with the three ocular disorders examined. These results suggest that the associations between these exposures and ocular outcomes are unlikely to be driven by shared genetic pleiotropy.

### 3.3. Results of MVMR Analysis

**Figure 2:**
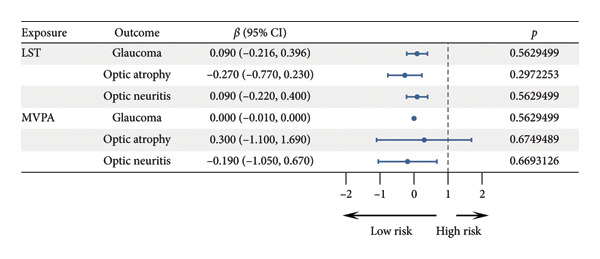
Forest plot illustrating the causal effects of leisure screen time (LST) and moderate‐to‐vigorous physical activity (MVPA) on optic nerve injury.

MVMR analysis was performed to evaluate the independent causal effects of LST and MVPA on ocular disorders, while simultaneously adjusting for multiple correlated exposures. In contrast to the univariable results, the MVMR analysis revealed significant associations after accounting for potential confounders (Figure 2).

Given that confounding factors might mask true associations in univariable analysis, we proceeded to MVMR analysis to assess independent effects after adjusting for key covariates including BMI, blood glucose, lipids, smoking, and alcohol consumption. Analysis of glaucoma risk factors indicated that, after adjusting for MVPA, alcohol consumption, BMI, blood glucose, blood lipids, and smoking, LST showed a significant positive association with glaucoma risk (*β* = 0.016, 95% CI: 0.004–0.027, *p* = 0.014), suggesting that sedentary behavior is an independent risk factor for glaucoma. Meanwhile, MVPA was significantly inversely associated with glaucoma risk (*β* = −0.012, 95% CI: −0.022 to −0.002, *p* = 0.026), indicating a protective role of PA. Additionally, smoking was also significantly associated with glaucoma risk (*β* = −0.020, 95% CI: −0.039 to −0.002, *p* = 0.037).

**Figure 3:**
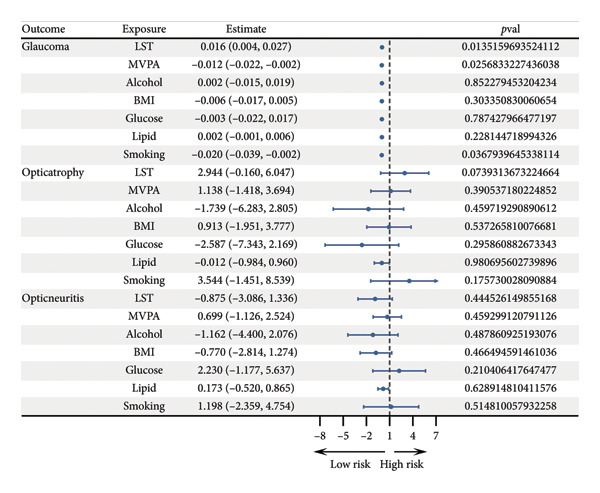
Forest plot of the multivariate analysis of the effects of leisure screen time (LST), moderate‐to‐vigorous physical activity (MVPA) on alcohol, body mass index (BMI), glucose, lipid, and smoking.

No significant associations were observed in the multivariable‐adjusted model with optic atrophy and optic neuritis. Sensitivity analyses supported the reliability of the MVMR findings. Multivariable Cochran’s *Q* test detected no significant heterogeneity (*p* > 0.05). The intercept test in MVMR‐Egger regression indicated no substantial horizontal pleiotropy (*p* > 0.05), suggesting that the results were not biased by genetic pleiotropy. Leave‐one‐out analysis confirmed that no single SNP unduly influenced the multivariable estimates, underscoring the robustness of the findings (Figure 3).

In summary, MVMR analysis revealed significant independent causal associations of both LST and MVPA with glaucoma risk after comprehensive adjustment for confounders, with LST acting as a risk factor and MVPA as a protective factor.

### 3.4. Gene Enrichment Analysis

To elucidate the potential mechanisms underlying the observed causal relationships, we performed GO and KEGG pathway analyses using the DAVID database on genes mapped from SNPs associated with LST, MVPA, and the three optic neuropathy–related traits. The results were visualized to interpret functional enrichment patterns.

KEGG enrichment analysis suggested that LST may influence the risk of optic nerve injury primarily through pathways related to insulin resistance, glucagon signaling, and adipocytokine activity, all of which are closely linked to impaired glucose and lipid metabolism. Consistently, GO analysis under biological process (BP) terms indicated significant enrichment in neurodevelopment, DNA damage response, inflammatory cytokine production, chylomicron remnant clearance, fatty acid clearance, and protein localization to the plasma membrane. Within cellular component (CC) categories, chylomicron remnants showed the strongest enrichment. For molecular function (MF), genes were notably enriched in the regulation of phosphatidylcholine‐sterol O‐acyltransferase activity.

**Figure 4:**
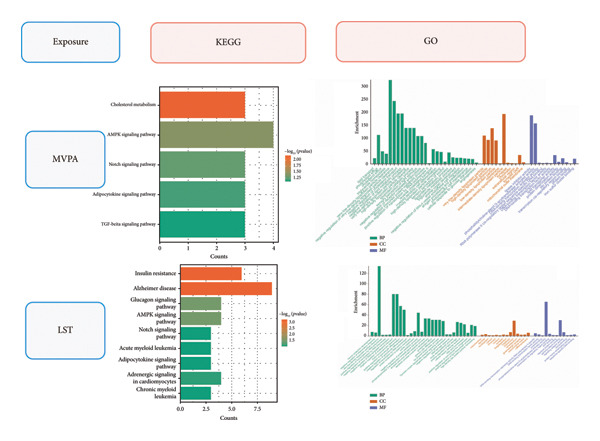
Gene enrichment of leisure screen time (LST) and four SNPs characteristic of optic nerve injury.

KEGG analysis suggested that MVPA was involved in cholesterol metabolism and adipocytokine‐related pathways. Interestingly, MVPA was also enriched in the TGF‐β signaling pathway, which is closely associated with fibrotic processes. In the GO analysis, BPs such as adipokine transport, cholesterol metabolism, and cellular response to high glucose and starvation were significantly enriched. CCs related to low‐ and very‐low‐density lipoprotein metabolism, chylomicron particles, and synaptic cleft were among the most enriched terms. MFs were dominated by lipase inhibitor activity and tau protein binding (Figure 4).

Additionally, LST may contribute to optic neuropathy through pathways linked to Alzheimer’s disease, AMPK signaling, and Notch signaling. In contrast, MVPA appears to modulate fibrotic pathways and cholesterol metabolism, potentially reducing the risk of optic nerve injury, particularly in glaucoma.

## 4. Discussion

Our findings provide genetic evidence that MVPA functions as a protective factor, whereas LST represents an independent risk factor for glaucoma, a neurodegenerative disorder characterized by optic nerve damage. Among these, UVMR results showed no significant direct associations between leisure screen time or MVPA and optic nerve injury. However, MVMR analysis suggested that after controlling for confounders, MVPA might reduce the risk of glaucoma, while leisure screen time might increase the risk. Moreover, smoking substantially amplifies the impact of these lifestyle behaviors on disease risk. These findings carry important clinical and public health implications. Supported by genetic evidence, they suggest that increasing PA and reducing prolonged sedentary behavior not only promote general health but may also provide a cost‐effective strategy for preventing glaucoma and other optic neuropathies. For individuals with a family history of glaucoma or additional risk factors, lifestyle modification may represent a critical component of primary prevention.

Mechanistically, LST may compromise optic nerve health through multiple pathways. Sedentary behavior is strongly linked to insulin resistance, dyslipidemia, and systemic inflammation [24]. Consistent with these associations, our enrichment analysis revealed that LST significantly influences chylomicron clearance [25], fatty acid metabolism [26], and neuroinflammatory processes [27], which may collectively aggravate microvascular injury and energy deficits in optic nerve neurons. Notably, LST‐related genes are enriched in Alzheimer’s disease pathways as well as the AMPK and Notch signaling pathways, suggesting that prolonged sedentary behavior may alter cellular proliferation and cell‐cycle dynamics, promoting neurodegenerative mechanisms [28, 29]. Nevertheless, the enrichment analysis results only suggest potential mechanisms, and their specific roles require validation in future studies. This potential shared pathological basis could also underlie the apoptosis of retinal ganglion cells in optic nerve damage [30]. Therefore, in clinical practice, individuals with prolonged sedentary behavior should be monitored not only for metabolic complications but also for an elevated risk of optic nerve damage.

In contrast, MVPA exerts a protective effect by improving lipid metabolism and inhibiting fibrosis [31]. We found that MVPA is significantly associated with cholesterol metabolism, adipokine signaling pathways, and the TGF‐β pathway, all of which are associated with organ fibrosis [32, 33], suggesting that exercise may reduce the risk of both intraocular pressure–dependent and independent optic nerve damage by optimizing lipoprotein metabolism, decreasing harmful lipid deposition [34], and inhibiting TGF‐β‐mediated optic nerve lamina cribrosa fibrosis [35]. Additionally, MVPA shows a positive effect on synaptic function and tau‐related pathways, further supporting its role in maintaining neuronal cell integrity [36]. This provides a theoretical foundation for future clinical trials based on exercise interventions, such as designing personalized exercise prescriptions for early glaucoma patients.

Moreover, smoking was found to modify the effects of LST and MVPA on glaucoma risk [37]. The detrimental impact of sedentary behavior appeared more pronounced in smokers, whereas the protective benefits of PA were stronger. These findings underscore the need to account for interactions between smoking and lifestyle behaviors in clinical guidance and risk communication [38]. We further support a comprehensive intervention model that integrates smoking cessation, increased PA, and reduced sedentary time to maximize the prevention of irreversible optic nerve damage.

Despite these findings, this study has several limitations. First, the GWAS data used primarily come from European populations, which limits the generalizability of the conclusions to other ethnic groups [39]. Second, while MR effectively reduces confounding, it cannot fully eliminate bias due to pleiotropy. Finally, the results of the enrichment analysis suggest potential biological mechanisms, but the specific roles of these pathways in optic neuropathy require further validation through basic and clinical research [40]. Future studies should incorporate more diverse population data and integrate prospective intervention designs to provide higher level evidence for the application of lifestyle factors in the prevention and treatment of optic nerve damage.

## 5. Conclusion

In conclusion, this study provides genetic evidence that MVPA is protective against glaucoma and other optic neuropathies, whereas LST independently increases their risk. Smoking further amplifies these lifestyle effects. Mechanistically, LST may contribute to disease development through disrupted lipid metabolism, neuroinflammation, and tau‐related neurodegenerative pathways, while MVPA exerts protective effects by enhancing cholesterol metabolism and suppressing TGF‐β‐mediated fibrosis. These findings underscore the importance of lifestyle modification as a cost‐effective strategy for the prevention and management of optic nerve disorders.

## Ethics Statement

This study utilized publicly available summary‐level data. All original data sources had obtained ethical approval and participant informed consent in their respective source studies.

## Conflicts of Interest

The authors declare no conflicts of interest.

## Funding

This work was supported by the Shanghai Natural Science Foundation (25ZR1401326), the Shanghai Healthcare System Key Discipline Construction Program (2024ZDXK0041), and the Shanghai Yangpu District Science and Technology Commission and Health Commission Program (YPM202302).

## Supporting Information

Supporting Table 1: STROBE‐MR checklist of recommended items to address in reports of Mendelian randomization studies.^1,2^

## Supporting information


**Supporting Information** Additional supporting information can be found online in the Supporting Information section.

## Data Availability

The data that support the findings of this study are available from the corresponding author upon reasonable request.
